# The phosphorylation status of VASP at serine 322 can be predictive for aggressiveness of invasive ductal carcinoma

**DOI:** 10.18632/oncotarget.4965

**Published:** 2015-08-12

**Authors:** Heike Döppler, Ligia Bastea, Sahra Borges, Xochiquetzal Geiger, Peter Storz

**Affiliations:** ^1^ Department of Cancer Biology, Mayo Clinic Comprehensive Cancer Center, Mayo Clinic, Jacksonville, FL 32224, USA; ^2^ Pathology and Laboratory Medicine, Mayo Clinic, Jacksonville, FL 32224, USA

**Keywords:** breast cancer, invasive, phosphorylation, VASP

## Abstract

Vasodilator-stimulated phosphoprotein (VASP) signaling is critical for dynamic actin reorganization processes that define the motile phenotype of cells. Here we show that VASP is generally highly expressed in normal breast tissue and breast cancer. We also show that the phosphorylation status of VASP at S322 can be predictive for breast cancer progression to an aggressive phenotype. Our data indicate that phosphorylation at S322 is gradually decreased from normal breast to DCIS, luminal/ER+, HER2+ and basal-like/TN phenotypes. Similarly, the expression levels of PKD2, the kinase that phosphorylates VASP at this site, are decreased in invasive ductal carcinoma samples of all three groups. Overall, the phosphorylation status of this residue may serve as an indicator of aggressiveness of breast tumors.

## INTRODUCTION

Members of the Ena/VASP protein family, such as Mammalian Enabled (Mena), Vasodilator-stimulated phosphoprotein (VASP) and Ena-VASP-like (EVL) have been linked to many human diseases [1]. In human breast cancer Mena and EVL have been shown to be regulated at the transcriptional level contributing to invasiveness. Mena is overexpressed in over 70% of primary breast cancers [2] and in benign lesions that have high risk of transformation [3]. Moreover, overexpression of Mena decreases overall patient survival in HER2-positive cancers [4]. Recent data demonstrate that alternately-spliced isoforms of Mena drive tumor cell invasion [5–7]. On the other hand, EVL-1 (a splice variant of EVL) seems to suppress the invasive phenotype in breast cancer by increasing actin bundling and decreasing protrusive activity of cells [5]. Subsequently, decreased expression of EVL-1 correlates with poor patient outcome due to high invasiveness [5]. In contrast to Mena and EVL-1, VASP expression levels do not serve as good predictive tumor markers, since they are generally high in both normal tissue and cancer. Another issue is that VASP can be phosphorylated at multiple sites, and depending on its phosphorylation status, may act as a promoter or inhibitor of tumor progression [8].

VASP function is defined by localization, tetramerization and different phosphorylation events [8, 9]. Several phosphorylated tyrosine, serine and threonine residues have been identified by mass spectroscopy (www.phosphosite.org), and so far five phosphorylation sites (Y39, S157, S239, T278 and S322) have been experimentally-confirmed and linked to cellular outcome [8]. Phosphorylation at S157 generally seems to mediate membrane localization of VASP [10, 11]. Although phosphorylation of VASP at S157 has been suggested as a marker for the potential of metastatic progression of prostate cancer [12], using this phosphorylation as a marker needs to be pursued with caution, since additional phosphorylations at S239/T278 or S322 can diverge the functions of VASP at the leading edge of migrating cells [8]. This can lead to opposite effects on the cell's potential to migrate, invade and metastasize. Phosphorylations at S239/T278 decrease F-actin accumulation and bundling [10, 13], while the phosphorylation at S322 increases F-actin accumulation [11, 14]. Moreover, the cells’ propensity to increase F-actin accumulation in response to VASP phosphorylation at S322 leads to decreased cell migration [11].

We here tested if phosphorylation of VASP at S157 and S322, two residues that have been shown to be phosphorylated by Protein Kinase D (PKD) and increase F-actin accumulation, can be indicative for invasive breast cancers. We found that phosphorylation of endogenous VASP in is mainly mediated by PKD2, and that active PKD2 mainly phosphorylates VASP at S322. Analyses of human samples indicate that both, decrease of PKD2 or phosphorylation of VASP at S322 may be predictive for an aggressive phenotype.

## RESULTS

### The expression level of VASP is not predictive for breast cancer survival or subtype

**Figure 1:**
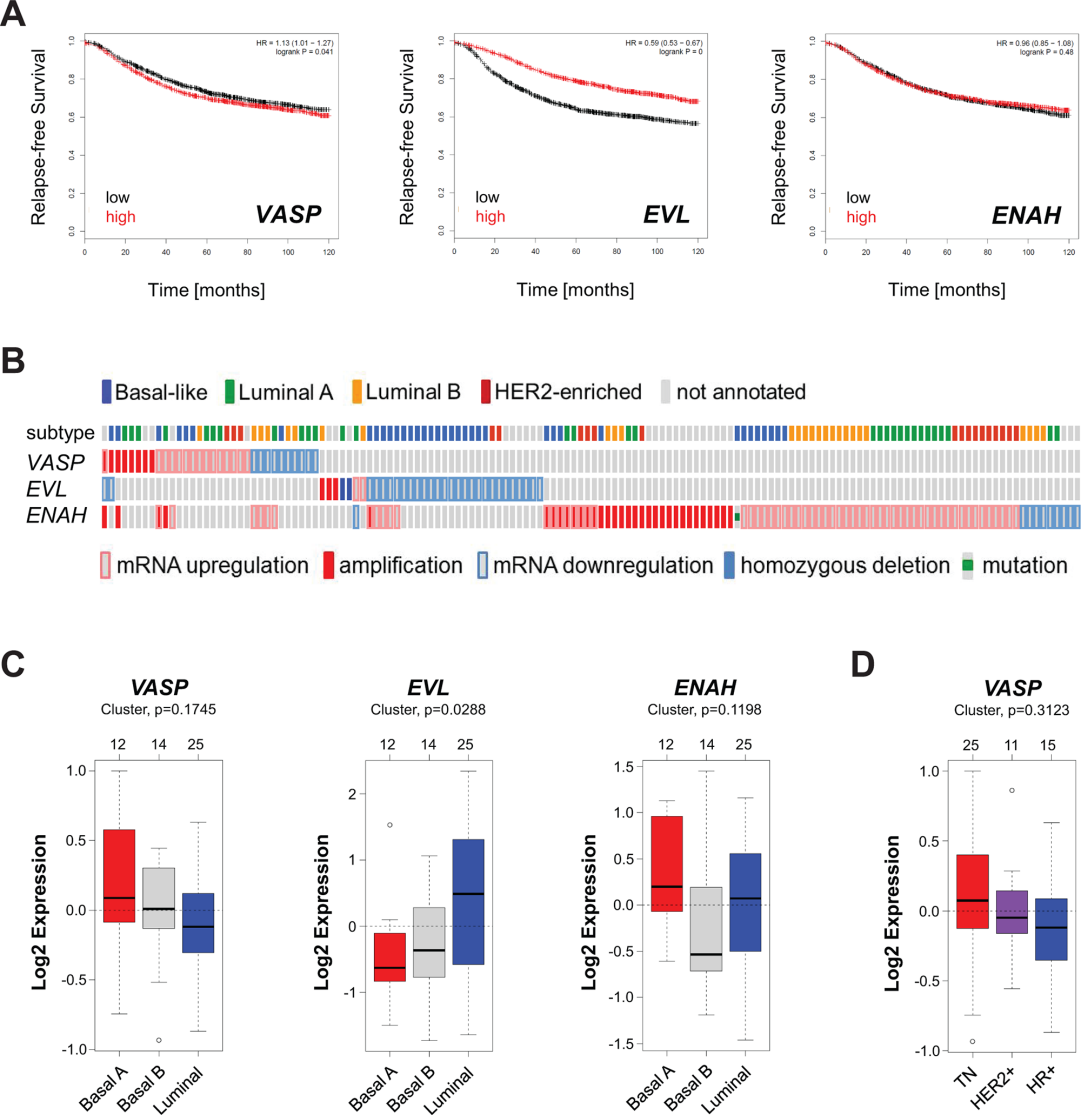
The expression of VASP is not predictive for breast cancer survival or subtype **A.** Relapse-free survival (RFS) of breast cancer patients with high or low expression of *VASP*, *EVL* and *ENAH* over time. The analysis was performed with the Kaplan-Meier Plotter (http://kmplot.com/analysis/index.php?p=service&cancer=breast) using standard settings. Patient samples (*n* = 3455) were split by median, the follow up threshold was set 10. **B.** Mutation or alterations in expression of *VASP*, *EVL* and *ENAH* in breast cancer. The analysis was performed using cBioPortal (http://www.cbioportal.org/public-portal/index.do). **C.** Relative expression of *VASP*, *EVL* and *ENAH* in breast cancer cell lines (*n* = 51) of basal or luminal subtypes. The analysis was performed using GOBO from Lund University (http://co.bmc.lu.se/gobo/). **D.** Relative expression of *VASP* in breast cancer cell lines (*n* = 51) grouped in TNBC, HER2+ or HR+ subtypes. The analysis was performed using GOBO from Lund University (http://co.bmc.lu.se/gobo/).

To determine if the expression levels of VASP can be predictive for breast cancer patient survival we analyzed a set of 3455 patient samples for which gene expression data was available. Samples were split by median and relapse-free survival (RFS) plotted over time. The analysis was performed using the Kaplan-Meier Plotter (http://kmplot.com/analysis/index.php?p=service &cancer=breast), previously described [15]. Patients with high expression of *VASP* had a slight decrease in RFS (Fig. 1A), which was statistically significant (*p* = 0.041). In addition we analyzed the two other Ena/VASP family members *EVL* (encodes EVL) and *ENAH* (encodes Mena). While high expression of *EVL* reversely correlated with patient RFS, the expression level of *ENAH* was not indicative of patient survival (Fig. 1A). Further analysis of mutations or alterations in breast cancer samples using cBioPortal (http://www.cbioportal.org/public-portal/index.do) [16, 17] indicated that *EVL* expression is downregulated (homozygous deletion or mRNA downregulation) mainly in basal-like and HER2 enriched tumors; *ENAH* expression is increased by mRNA upregulation or gene amplification and this was not specific to a breast cancer subtype (Fig. 1B). Of the few tumors with VASP alterations approximately one third showed downregulation of mRNA and two thirds an upregulation of expression (mRNA level or DNA amplification). These alterations did not correspond to any specific breast cancer subtype (Fig. 1B).

We next analyzed a set of 51 cell lines for altered expression of Ena/VASP family members between luminal and basal phenotypes of breast cancer using GOBO from Lund University (http://co.bmc.lu.se/gobo/) [18]. While differences of expression of *VASP* and *ENAH* were not significant between subtypes, expression of EVL was significantly higher in the less aggressive luminal subtype (Fig. 1C). In addition, for VASP, we grouped cell lines into TN, HER2 positive or HR positive groups, but also did not observe significant differences in its expression between subtypes (Fig. 1D). Overall, these data suggest that VASP expression levels are not indicative for the subtype of breast cancer and are not suitable predictive markers for patient survival. They also confirm that decreased expression of EVL is a feature of the aggressive basal phenotype. However, they also suggest that *ENAH*, while often upregulated, cannot be correlated with aggressiveness.

### The phosphorylation status of VASP at S322 indicates aggressiveness of invasive ductal carcinoma

**Figure 2:**
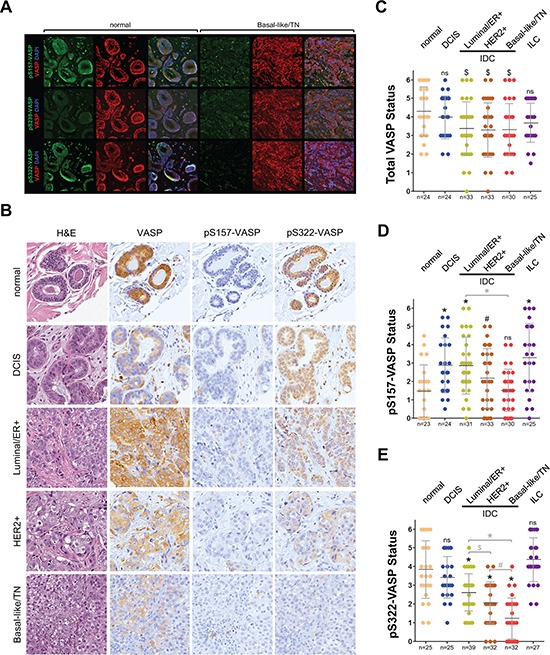
Phosphorylation of VASP at S157 and S322 is decreased in triple-negative breast cancer **A.** Immunofluorescence staining of serial sections of normal breast tissue or TNBC. Samples were stained for pS157-VASP (top row, green), pS239-VASP (middle row, green), or pS322-VASP (bottom row, green), as well as total VASP (red) and nuclei using DAPI (blue). Shown are representative areas of a representative sample from *n* = 5 samples analyzed per group. **B-E** Tissue microarrays with indicated groups of samples were immunohistochemically-stained for total VASP, pS157-VASP or pS322-VASP. Relative expression was determined and rated from 0–6 (0 = no expression; 6 = strongest expression). B shows H&E and IHC staining of samples of each group representing the average readout signal observed. C–D show quantitation analyses of TMAs as described in Materials & Methods. * in black = *p* < 0.0001 as compared to normal tissue; * in grey = *p* < 0.0001 as compared to indicated group; # in black = *p* < 0.005 as compared to normal tissue; # in grey = *p* < 0.005 as compared to indicated group; $ in black = *p* < 0.05 as compared to normal tissue; $ in grey = *p* < 0.05 as compared to indicated group; ns = not significant as compared to normal tissue.

Next, we tested if VASP phosphorylation at S157, S239 or S322 is altered during progression of breast cancer. Therefore, we first compared samples of normal breast tissue to triple negative breast cancer (TNBC). Immunofluorescence analysis of total VASP indicated high expression in both normal tissue and TNBC (Fig. 2A, red immunofluorescence). Phosphorylation at S157 showed patchy staining in normal and TNBC, with high variations in different samples (not shown), whereas S239 phosphorylation was low in both (Fig. 2A, rows one and two, green fluorescence). The most significant difference was observed when we probed for S322 phosphorylation, which was high in normal and low, almost absent in TNBC (Fig. 2A, row three, green fluorescence). Since the immunofluorescence data indicated that changes in the status of S322 (and possibly S157, although patchy) could be predictive for a progression of breast cancer to a more aggressive and invasive phenotype we next analyzed a larger set of patient samples for these two phosphorylations. Immunohistochemical (DAB staining) analysis of progression tissue microarrays (TMAs) including normal breast tissue, ductal carcinoma *in situ* (DCIS), 3 groups of invasive ductal carcinomas (ER positive; HER2 positive; or TN) indicated that levels of total VASP are not indicative for progression (Figs. 2B and 2C). We also did not observe a clear trend between samples for phosphorylation at S157. There even was a slight, but significant increase in phosphorylation in DCIS, ER+ IDC, HER2+ IDC and ILC (Fig. 2D). Analysis of phosphorylation at S322 indicated a clear trend. We found that all samples of IDC showed significantly less phosphorylation at this site (Fig. 2E), whereas levels in DCIS and ILC were comparable to normal tissue. Within the group of IDC, we observed a gradual decrease in S322 phosphorylation with increasing aggressiveness of breast cancers (compare ER+ to HER2+ to TN groups).

### Mimicking S157 and S322 phosphorylations in HuMEC inhibits directed cell migration

**Figure 3:**
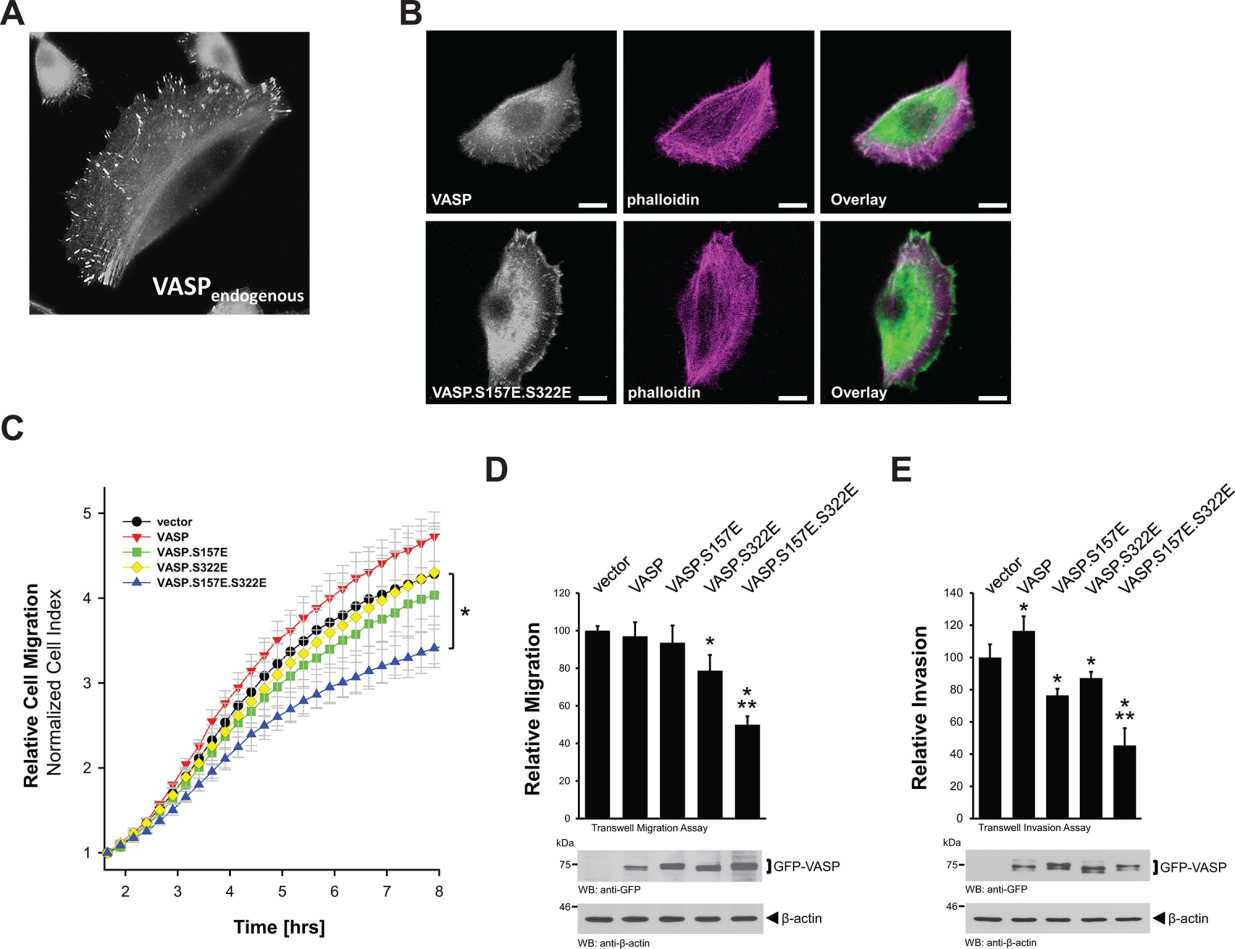
Mimicking S157 and S322 phosphorylations inhibits directed cell migration **A.** HuMEC cells were fixed and stained for endogenous VASP by immunofluorescence. **B.** HuMEC cells were transfected with GFP-tagged VASP or VASP.S157E.S322E. After 24 hours cells were fixed and F-actin was stained with phalloidin. The localization of GFP-tagged proteins was determined using immunofluorescence analysis (bar is 10 μm). **C.** HuMEC cells were transfected with vector control, GFP-tagged versions of VASP, VASP.S157E, VASP.S322E, or VASP.S157E.S322E. Real-time directed cell migration towards NIH3T3-conditioned media was monitored in impedance-based transwell assays (xCELLigence RTCA DP system, Transwell CIM-plates 16 from Roche) over a time period of 12 hours. The asterisk indicates statistical significance. **D, E.** MDA-MB-231 cells were transfected with vector control, GFP-tagged versions of VASP, VASP.S157E, VASP.S322E, or VASP.S157E.S322E. Transwell assays were performed as described in Materials & Methods to determine cell migration or cell invasion towards NIH3T3-conditioned media. * indicates statistical significance as compared to the vector control. ** indicates statistical significance as compared to single mutants. Equal expression of overexpressed GFP-tagged VASP or mutants was controlled by Western blotting for GFP (anti-GFP). Staining of lysates for β-actin served as loading control.

We recently have shown for HeLa cells that phosphorylation at S157 and S322 drives VASP from focal contacts to the leading edge, which results in a decrease in cell migration [11]. We also have shown that both phosphorylations can be mimicked with serine to glutamate mutations at these sites [11]. In human mammary epithelial cells (HuMEC), endogenous VASP mainly is localized at the focal contacts (Fig. 3A). Mimicking phosphorylations at S157 and S322 in HuMEC also resulted in increased localization of VASP at the leading edge (Fig. 3B) and decreased directed cell migration (Fig. 3C). While phosphorylation at S157 is necessary for membrane localization [10], phosphorylation at S322 regulates actin reorganization processes once VASP is located to the membrane [11]. Therefore, a downregulation of both phosphorylations may be required for invasive cells to increase motility. Indeed, our data using single mutants (Fig. 3C) indicate that both phosphorylations are required in order to efficiently block migration. Similar additive effects of both phosphorylations on cell migration and cell invasion were also observed in highly motile MDA-MB-231 breast cancer cells (Figs. 3D and 3E).

### Phosphorylation of endogenous VASP at serines 157 and 322 is mediated by PKD2

**Figure 4:**
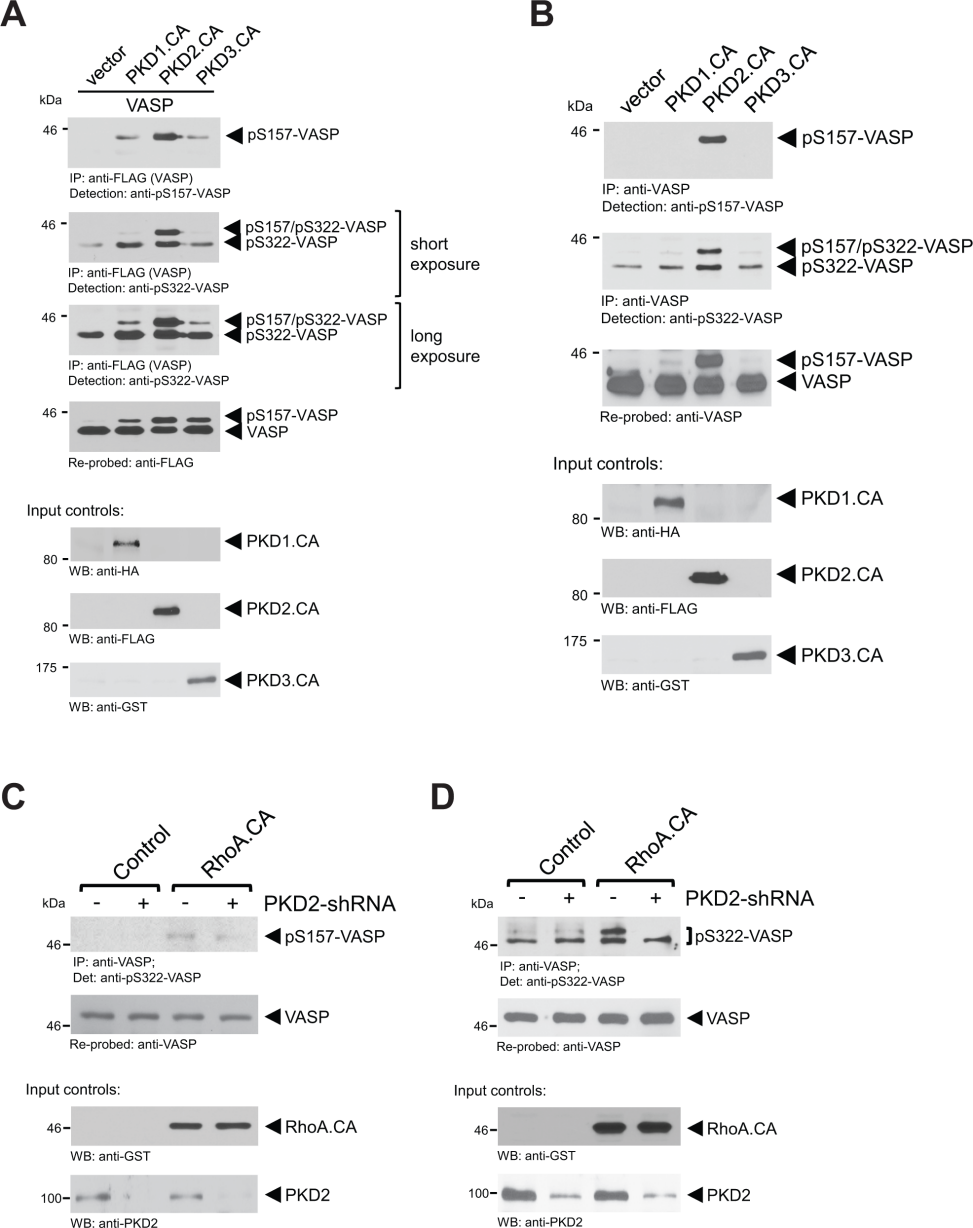
Phosphorylation of endogenous VASP at S157 and S322 is mediated by PKD2 **A.** Cells were transfected with constitutively-active versions of PKD1, PKD2 or PKD3 together with FLAG-tagged VASP. Cells were lysed, overexpressed VASP was immunoprecipitated (anti-FLAG), and immunoprecipitates were analyzed by SDS-PAGE & immunoblotting for indicated phosphorylations using phospho-specific antibodies. Samples were re-probed for total VASP by staining with anti-FLAG. In addition expression of active PKD isoforms was determined by Western blotting of lysates with tag-specific antibodies as indicated. **B.** Cells were transfected with constitutively-active versions of PKD1, PKD2 or PKD3. Cells were lysed, endogenous VASP was immunoprecipitated (anti-VASP) and immunoprecipitates were analyzed by SDS-PAGE and immunoblotting for indicated phosphorylations using phospho-specific antibodies. Samples were re-probed for endogenous total VASP. Expression of active PKD isoforms was determined by Western blotting of lysates with tag-specific antibodies as indicated. **C, D.** Cells were co-transfected with control vector or PKD2-shRNA and vector or constitutively-active RhoA, as indicated. Endogenous VASP was immunoprecipitated (anti-VASP) and immunoprecipitates were analyzed by SDS-PAGE and immunoblotting for indicated phosphorylations using phospho-specific antibodies. Samples were re-probed for endogenous total VASP. Lysates were probed by Western blotting for expression of active RhoA (anti-GST) or for expression of PKD2 (anti-PKD2).

Previously, we have identified PKD1 as a kinase that, when ectopically-expressed in cells, phosphorylates S157 and S322 [11]. In order to determine if there is a preference of one of the three PKD isoforms for VASP as a substrate, we first ectopically-expressed active versions of all three isoforms together with FLAG-tagged VASP in cells and analyzed for phosphorylations. Surprisingly, as compared to active PKD2, active alleles of PKD1 and PKD3 only led to a weak phosphorylation of VASP at S157 and S322 (Fig. 4A). This suggested that of the PKD family members, PKD2 may be the kinase that mediates phosphorylation of VASP at both sites under endogenous conditions. To test this we again expressed active versions of all three PKD isoforms, but immunoprecipitated and analyzed endogenous VASP. Our data clearly indicate that phosphorylation of endogenous VASP at both residues (S157 and S322) is a PKD2-specific event (Fig. 4B). However, introducing an active mutant of RhoA to activate PKD2, led to only weak phosphorylation of endogenous VASP at S157 (Fig. 4C), but a significant increase in S322 phosphorylation (Fig. 4D). Both RhoA-induced phosphorylations were decreased in presence of PKD2-shRNA.

### Phosphorylation of VASP at S322 is decreased in metastatic breast cancer cells

**Figure 5:**
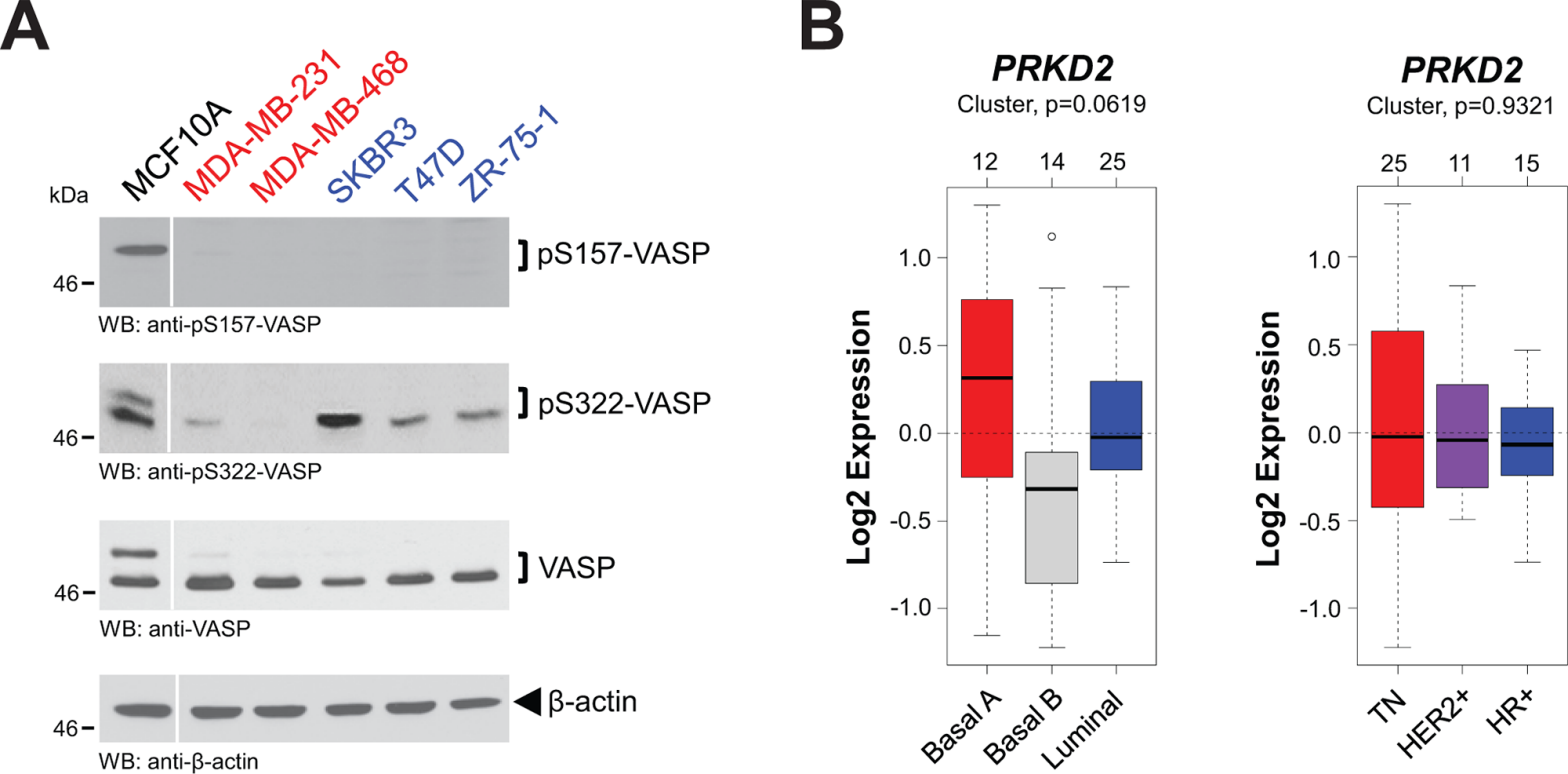
Expression and phosphorylation status of VASP and PKD2 **A.** Lysates of indicated cell lines were analyzed by Western blot for expression of endogenous VASP and its phosphorylations at S157 or S322. Labeled in red are cell lines that are highly-invasive; in blue are cell lines that are little invasive. Staining for β-actin served as an additional loading control. **B.** Relative expression of *PRKD2* in breast cancer cell lines (*n* = 51) of basal or luminal subtypes or grouped in TNBC, HER2+ or HR+ subtypes. The analysis was performed using GOBO from Lund University (http://co.bmc.lu.se/gobo/).

Next we analyzed a panel of breast cancer cell lines, containing highly-invasive cell lines (MDA-MB-231 and MDA-MB-468) that are metastatic in animal models, as well as hormone receptor-positive cell lines (ZR-75–1, T47D and SKBR3) that are not metastatic *in vivo*, for VASP phosphorylations. MCF10A cells served as a positive control for VASP phosphorylations. We found that under normal growth conditions in all breast cancer cell lines, VASP was only marginally or not phosphorylated at S157. Phosphorylation of S322, however, reversely correlated with their metastatic behavior, and was increased in cells that are not forming metastases in mice (Fig. 5A). In all these cell lines, PKD2 generally was expressed and not indicative for VASP phosphorylations (not shown). Therefore, we performed additional analysis of the 51 cell lines in the GOBO database for PKD2 expression, but also did not detect statistically-significant differences between triple-negative or hormone receptor positive cell lines, nor when cells were classified into basal and luminal subtypes (Fig. 5B).

### The expression level of PKD2 is predictive for breast cancer subtype and relapse-free survival

**Figure 6:**
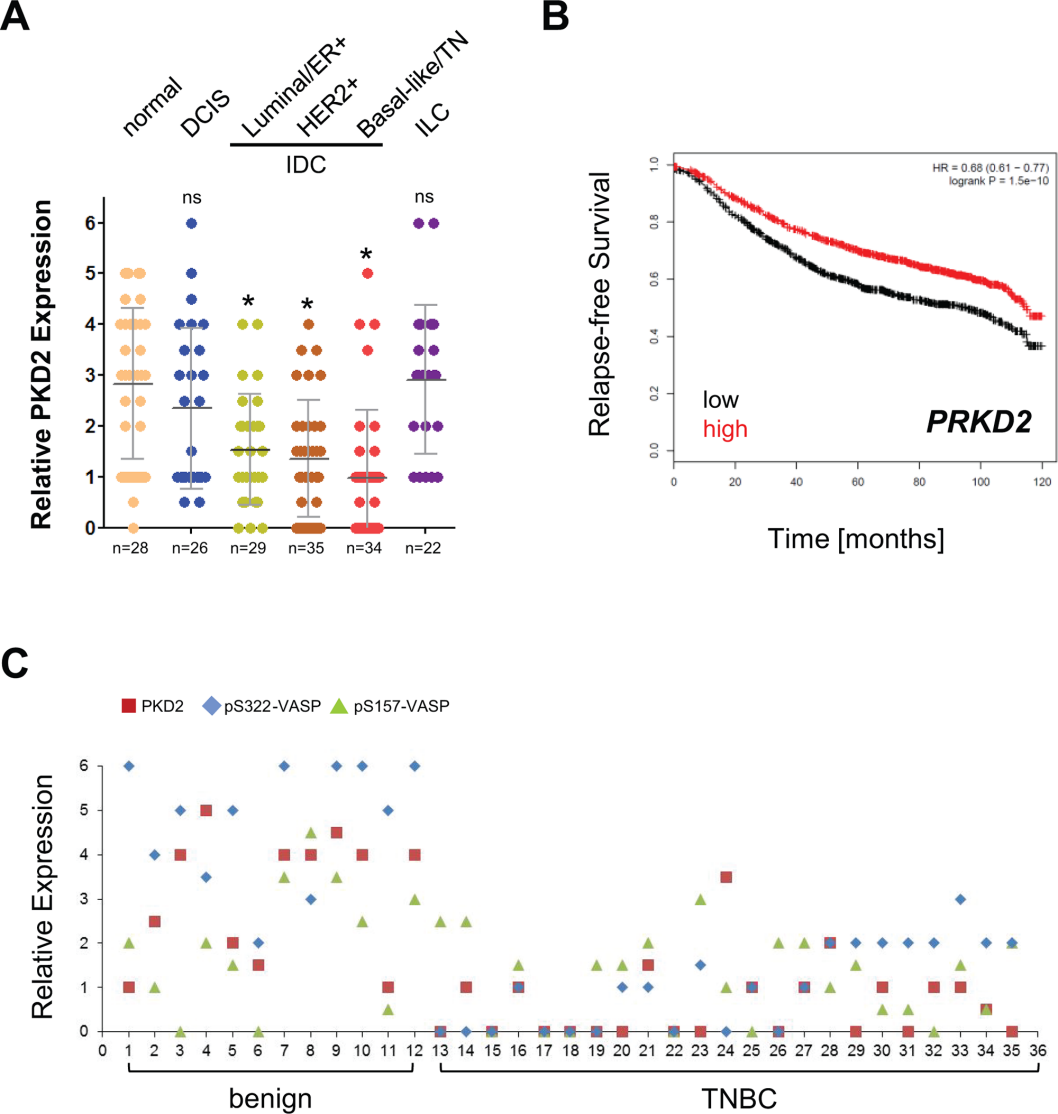
The expression of the S157/S322 kinase PKD2 is decreased in IDC and relative expression levels predict RFS **A.** Tissue microarrays with indicated groups of samples were immunohistochemically-stained for PKD2. Relative expression was determined and rated from 0–6 (0 = no expression; 6 = strongest expression). * in black = *p* < 0.0001 as compared to normal tissue; ns = not significant as compared to normal tissue. **B.** Relapse-free survival (RFS) of breast cancer patients with high or low expression of *PRKD2* over time. The analysis was performed using the Kaplan-Meier Plotter (http://kmplot.com/analysis/index.php?p=service&cancer=breast) using standard settings. Patient samples (*n* = 3455) were split by median, the follow up threshold was set 10. **C.** Relative expression of PKD2, pS322-VASP and pS157-VASP in benign and TNBC patient samples. The quantitation analysis of TMAs is described in Materials & Methods.

We next tested our set of patient sample TMAs for PKD2 expression. Similar as observed for VASP phosphorylation at S322, we found that PKD2 levels are significantly decreased in IDC (Fig. 6A). This result was somewhat surprising, since in a previous study we did not notice this difference in PKD2 levels, most likely because of a too low power for normal samples (*n* = 10 in [19] as compared to *n* = 28 in Fig. 6A). Eventually, to determine if PKD2 expression levels in breast cancer can be predictive for relapse-free survival, we analyzed a set of 3455 patient samples using the Kaplan-Meier Plotter (http://kmplot.com/analysis/index.php?p=service &cancer=breast), previously described [15]. Samples were split by median and relapse-free survival (RFS) plotted over time. As expected patients with high expression of *PRKD2* showed an increase in RFS that was statistically extremely significant (Fig. 6B). A direct comparison of matching patient tissue showed a concordance between PKD2 expression and VASP phosphorylations at S157 and S322 in benign tissue and TNBC. In TNBC, low expression of PKD2 correlated with decreased phosphorylation of VASP (Fig. 6C).

## DISCUSSION

VASP is a critical switch downstream of RhoGTPases that controls dynamic actin reorganization processes that define the motile phenotype of cells [20, 21]. For example, VASP regulates F-actin filament elongation and bundling and contributes to cell-cell adhesions, focal adhesions and filopodia formation [22–24]. The expression levels of the Ena/VASP protein family members Mena and EVL-1 have been linked to patient outcome in breast cancer, whereas VASP has been described as expressed in relatively high levels in normal as well as cancer tissue. Only in lung adenocarcinomas increased VASP expression was described when compared to normal epithelium [25]. However, in cell culture experiments homozygous knockout of VASP suggested rather an inhibitory role for VASP on soft agar colony and tumor formation [26]. Here, we used several available databases to determine if VASP expression in breast cancer can be linked to patient survival or tumor type. Initially, as control, we included the two other family members Mena (*ENAH*) and EVL (*EVL*) into our studies. Mena, for example, has been described to be overexpressed in over 70% of primary breast cancers [2] and in invasive mammary tumor cells [27]. Moreover, it was suggested that high expression decreases overall patient survival in HER2-positive cancers [4]. While our analysis of alterations in breast cancer samples confirms that mRNA upregulation or gene amplification of Mena frequently occurs in all types of invasive breast cancer (Fig. 1B), Kaplan-Meier Plot analysis of a set of 3455 patients for relapse-free survival (Fig. 1A) did not suggest any correlation between Mena expression levels and patient survival. We also did not observe significant differences in expression of Mena in cell lines between luminal and basal phenotypes. However, it also was shown that alternative splicing of Mena is needed to produce an invasion-promoting isoform [6, 7], and the databases used do not allow us to distinguish between splice forms for our analysis. For EVL-1, a splice variant of EVL, it was previously shown that decreased expression correlates with poor patient outcome due to high invasiveness [5]. Like VASP, EVL-1 can be regulated by PKD through phosphorylation, however at a different residue [28]. In addition, although the phosphorylation of EVL-1 by PKD1 has been shown to occur in lamellipodia, filopodia and at cell-cell contacts, so far no information exists if it is predictive for aggressiveness of human breast cancer.

Like previously shown for EVL-1, our data now confirm for EVL that decreased expression correlates with poor patient outcome (Fig. 1), but also expand this and implicate that mRNA downregulation may mainly occur in samples or cell lines of the basal-like phenotype (Figs. 1B, 1C). The conclusion from our analyses is that levels of EVL expression can indeed serve as predictive marker for patient survival. For VASP we found only a marginal difference in relapse-free survival between patients with high or low expression (Fig. 1A). Similarly, in cell lines, the VASP expression status was not significantly different between the luminal or basal subtypes, nor when samples were grouped conventionally (TN, HER2+ or HR+).

A caveat of “only” looking at expression levels is that VASP can be phosphorylated at multiple sites with different outcome, and dependent on its phosphorylation pattern, VASP may act as a promoter or inhibitor of tumor progression [8]. Some attempts have been made to link phosphorylation of VASP at certain residues to tumor survival or progression. For example, S157 phosphorylation of VASP has been suggested as a marker for prostate cancer cell motility and potential of metastatic progression [12]. However, the phosphorylation status at S157 alone is not indicative for cancer cell motility. Phosphorylation of S157 drives VASP to the leading edge, but additional phosphorylations at S239/T278 or S322 bifurcate VASP functions in respect to F-actin accumulation, filopodia formation and cell migration. We here tested if these phosphorylations can serve as more conclusive markers for tumor aggressiveness. Due to the lack of appropriate antibody tools other phosphorylations of VASP (i.e. Y39) currently cannot be assessed for their use as predictive markers for breast cancer progression. Of these especially Y39 phosphorylation of VASP which is mediated by the tyrosine kinase Abl may be of interest [29, 30]. Because Abl is an upstream kinase for PKD [31, 32], the phosphorylation of Y39 may be functionally linked to S322 phosphorylation and expression patterns for pS322 and pY39 may overlap.

Phosphorylations of VASP at S239 and T278 are mainly mediated by PKA, PKG and AMPK [8]; and suppress motility in cancer cells and colon cancer [10, 33–35]. We did not detect significant changes in phosphorylation of VASP at S239 (Fig. 2A, and *data not shown* for analysis of TMAs) or T278 (not shown) when comparing normal breast tissue to patient samples. Therefore, we focused on phosphorylation at S322, which can be mediated by PKD enzymes (mainly PKD2 as demonstrated in Fig. 4) and (in combination with S157 phosphorylation) decreases directed cell migration [11]. At this point it is unclear if in invasive breast cancer cells and tissue VASP phosphorylation at both sites is exclusively regulated PKD2, since both also can be targeted by AMPK, a metabolic gauge associated with tumorigenesis [14].

PKD enzymes are downstream of RhoGTPases (discussed in [36]). PKD1 for example has been shown to be activated by RhoA, while it is negatively regulated by Rac-1 [8, 11, 37]. In addition to VASP several other signaling molecules that contribute to cell migration are targeted by RhoA-regulated activation of PKD enzymes [38–40]. However, although PKD2 also is regulated by RhoA (Fig. 4 and [41]), only little is known on its substrates.

Our data indicate that VASP expression levels cannot be used as predictive markers for type or outcome in breast cancer. The phosphorylation status at S322, however, correlates with aggressiveness and a gradual decrease is observed in IDC from ER+ to HER2+ to TN cancers, while it is not indicative for invasive lobular carcinoma (ILC). Overall, the phosphorylation status of this residue may serve as both an indicator of aggressiveness of invasive breast tumors of ductal origin.

## MATERIALS AND METHODS

### Antibodies, reagents, DNA constructs and cell lines

HuMEC cells (Invitrogen, Carlsbad, CA) were maintained in HMEC Culture System from Invitrogen. All other cells lines were obtained from the American Type Culture Collection (Manassas, VA). MDA-MB-231, HeLa and T47D cells were maintained in Dulbecco's modified Eagle's medium (DMEM) with 10% fetal bovine serum (FBS). ZR-75-1 and MDA-MB-468 were maintained in RPMI 1640 with 10% FBS. SKBR3 was maintained in McCoy's 5a Medium with 10% FBS. MCF-10A cells were maintained in DMEM/Ham's F-10 medium (50:50 vol/vol) with 5% horse serum, 20 ng/ml EGF, 0.5 μg/ml hydrocortisone, 100 ng/ml cholera toxin, 10 μg/ml insulin and 1% penicillin/streptomycin. EGF was from PeproTech (Rocky Hill, NJ), insulin and hydrocortisone from Sigma-Aldrich (St Louis, MO). Anti-β-actin antibody was from Sigma-Aldrich, anti-VASP antibody from BD Transduction Laboratories (San Jose, CA), anti-PKD2 antibody from Upstate Biotechnology (Charlottesville, VA). The phosphorylation-specific antibodies anti-pS239-VASP and anti-pS157-VASP antibodies were from Cell Signaling Technology (Danvers, MA). The anti-pS322-VASP antibody was made by 21^st^ Century Biochemicals (Marlboro, MA) and is further described in [11]. Secondary HRP-linked antibodies were from Millipore (Billerica, MA) and secondary antibodies for immunofluorescence (Alexa Fluor 488 F(ab’)2 fragment of goat-anti-rabbit IgG or Alexa Fluor 568 F(ab’)2 fragment of goat-anti-mouse) were from Invitrogen. TransIT HeLa Monster (Mirus Bio, Madison, WI) was used for transient transfection of HeLa, PolyJet (SignaGen Laboratories, Rockville, MD) for MDA-MB-231, and Lipofectamine 2000 (Invitrogen) for HuMEC cells. Expression plasmids for FLAG-tagged human VASP, GFP-tagged human VASP and phosphorylation mimicking (VASP.S157E, VASP.S322E, or VASP.S157E.S322E) mutants, as well as the expression constructs for tagged constitutively-active versions (S to E mutations in critical activation loop serines) of PKD1, PKD2 or PKD3 have been described in detail elsewhere [11, 40]. The expression construct for constitutively-active RhoA has been described in [38], and the PKD2-shRNA in [32].

### Immunoblotting, immunoprecipitation and SDS-PAGE

Cells were washed twice with cold (4°C) PBS (140 mM NaCl, 2.7 mM KCl, 8 mM Na_2_HPO_4_, 1.5 mM KH_2_PO_4_, pH 7.2). After lysis with lysis buffer (50 mM Tris-HCl pH7.4, 1% Triton X-100, 150 mM NaCl, 5 mM EDTA pH 7.4) plus Protease Inhibitor Cocktail (PIC, Sigma-Aldrich), samples were incubated on ice (30 min), centrifuged at 13,000 rpm (15 min, 4°C) and protein concentration was determined. Lysates then were either analyzed by Western blot or subjected to immunoprecipitation, as indicated. For immunoprecipitation, lysates were incubated with target-specific antibody (2 μg) for one hour, followed by incubation with protein G-Sepharose (GE Healthcare, Piscataway, NJ) for 30 minutes. Immune-complexes were washed 3 times with TBS (50 mM Tris-HCl pH 7.4, 150 mM NaCl), resolved in 20 μl TBS and 2x Laemmli buffer, subjected to SDS-PAGE, transferred to nitrocellulose membranes and visualized by immunostaining.

### Tissue microarrays (TMAs)

Tissue samples were initially collected with the approval of the Mayo Clinic Institutional Review Board (IRB) under protocol MC0033. Written informed consent for the use of these tissues in research was obtained from all participants. Generation of the TMA was performed under protocol 09–001642. Therefore, all unique patient identifiers and confidential data were removed and tissue samples were de-identified. The Mayo Clinic Institutional Review Board assessed the protocol 09–001642 as minimal risk and waived the need for further consent. Tissue microarrays (TMAs) including normal breast tissue (adjacent to tumor or from mammoplasty), ductal carcinoma *in situ* (DCIS), 3 groups of invasive ductal carcinomas (ER positive; HER2 positive; or TN) as well as invasive lobular carcinoma (ILC) have been described before [42]. In brief, representative areas of patient tissues/tumors were selected and marked by a breast pathologist. Representative 1.5 mm punches were performed, TMAs were generated, and presence of representative tissue was controlled after H&E staining. The TMAs were analyzed in duplicates. All were scored independently by two different experienced scientists. Uniform pre-established criteria were used. Immunoreactivity was graded semiquantitatively by considering the intensity of the staining of the ductal/tumor cells throughout the whole area. A histological score was obtained from each sample, which ranged from 0 (no immunoreaction) to 6 (maximum immunoreactivity as seen in normal ductal tissue). All samples of each group were included into the graphs shown. Reproducibility of the scoring method between three observers was greater than 90%. In the remaining cases, in which discrepancies had been noted, differences were settled by consensus review of corresponding tissues.

### Immunohistochemistry and immunofluorescence on tissues

Slides were de-paraffinized (60°C, one hour), de-waxed in xylene (five times for four minutes), rehydrated with ethanol (100%, 95%, 75%, twice with each concentration for three minutes) and then rinsed in water. The rehydrated TMAs were rinsed in water and subjected to antigen retrieval in citrate buffer (pH 6.0) as described by the manufacturer (DAKO, Carpinteria, CA, USA). After antigen retrieval in 10 mM sodium citrate buffer (pH 6.0), slides were treated with 3% H_2_O_2_ (five minutes) to reduce endogenous peroxidase activity, washed with PBS containing 0.5% Tween 20, and blocked with protein block serum-free solution (DAKO, Carpinteria, CA) for five minutes at room temperature. For immunohistochemistry, anti-VASP (1:2000), anti-pS157-VASP (1:25), anti-pS322-VASP (1:1000), or anti-PKD2 (1:1000) antibodies were diluted in Antibody Diluent Background Reducing Solution (DAKO) and visualized using the EnVision Plus Anti-Rabbit Labelled Polymer Kit (DAKO) according to the manufacturer's instructions. Images were captured using the ScanScope XT scanner and ImageScope software (Aperio, Vista, CA). For immunofluorescence, blocked sections were incubated with anti-VASP (1:2000), anti-pS157-VASP (1:100), anti-pS239-VASP (1:100), or anti-pS322-VASP (1:1000) antibodies in Antibody Diluent Background Reducing solution (Dako) at 4°C, overnight. After 3 washes with PBS containing 0.05% Tween-20, Alexa Fluor 488 or Alexa Fluor 568 labeled secondary antibodies from Invitrogen (Grand Island, NY) were added at a 1:500 dilution (RT, 1 hour). DAPI (final concentration 125 μg/ml) was added for 15 minutes after samples were incubated with the secondary antibodies. LabVision PermaFluor (Thermo Scientific) was used as mounting medium. Images were captured by a fluorescent scanner (ScanScope FL, Aperio) with consistent exposure time and processed using ImageScope software (Aperio).

### Immunofluorescence of cells

Cells were washed with PBS (twice), fixed with 4% paraformaldehyde (15 min, 37°C), washed with PBS (three times) and then permeabilized with 0.1% Triton X-100 in PBS (2 min, RT). Samples were blocked with 3% bovine serum albumin and 0.05% Tween 20 in PBS (blocking solution) for 30 min at RT, and then incubated over night at 4°C with primary antibody (anti-VASP 1:1000) diluted in blocking solution. Samples then were washed with PBS (five times) and incubated with secondary antibodies (Alexa Fluor 488 F(ab’)2 fragment of goat-anti-mouse IgG, Invitrogen), diluted (1:800) in blocking solution for 2 hours at RT. After extensive washes in PBS, cells were mounted in Ibidi mounting medium (Ibidi, Martinsried, Germany). When cells were transfected with GFP-tagged versions of VASP, transfection was performed in 8 well ibiTreat μ-Slides (Ibidi). After fixing and blocking steps, F-actin structures were stained with phalloidin (Alexa Fluor 633-Phalloidin, shown as magenta pseudocolor staining) in blocking solution. All samples were examined using an IX81 DSU Spinning Disc Confocal from Olympus with a 40x objective.

### Impedance-based real-time cell migration analysis

For real-time analysis of directed cell migration, cells were transfected as indicated and after 24 hours seeded on Transwell CIM-plate 16 plates (Roche, Indianapolis, IN). After 1.5 hours of attachment, cell migration towards NIH-3T3 conditioned media was monitored over 12 hours in real-time using the xCELLigence RTCA DP instrument (Roche).

### Transwell migration and invasion assays

Transwell migration and invasion assays were performed as described previously [38]. In short, transwells were left uncoated (migration assay) or coated with Matrigel (2 μg/well; BD Biosciences, San Jose, CA), dried overnight and rehydrated for 1 hour with 40 μl of tissue culture media. Cells were harvested, washed once with media containing 1% bovine serum albumin (BSA) and resuspended in media containing 0.1% BSA. 100,000 cells were seeded per transwell insert. NIH-3T3 conditioned medium served as a chemoattractant. Remaining cells were used to control the expression of genes of interest by Western blot. After 16 hours, cells on top of the transwell insert were removed and cells that had migrated/invaded to the lower surface of the filters were fixed in 4% paraformaldehyde. For analyses, GFP-expressing cells were counted and numbers were normalized to transfection efficiency.

### Statistical analysis

Data are presented as mean ± SD. *P* values were acquired with the student's *t*-test using Graph Pad software, and *p* < 0.05 was considered statistically significant.

## Abbreviations

DCIS: ductal carcinoma *in situ*
ER: estrogen receptor
EVL: Ena-VASP-like
F-actin: filamentous actin
HER: human epidermal growth factor receptor 2
HR: hormone receptor
IDC: invasive ductal carcinoma
ILC: invasive lobular carcinoma
Mena: Mammalian Enabled
PKD: protein kinase D
PKG: cGMP-dependent protein kinase
VASP: vasodilator-stimulated phosphoprotein
TN: triple-negative
